# Rapid kinetics of iron responsive element (IRE) RNA/iron regulatory protein 1 and IRE-RNA/eIF4F complexes respond differently to metal ions

**DOI:** 10.1093/nar/gku248

**Published:** 2014-04-09

**Authors:** Mateen A. Khan, Jia Ma, William E. Walden, William C. Merrick, Elizabeth C. Theil, Dixie J. Goss

**Affiliations:** 1 Department of Chemistry and Biochemistry, Hunter College, City University of New York, New York, NY 10065, USA; 2 Department of Microbiology and Immunology, University of Illinois at Chicago, Chicago, IL 60612-7334, USA; 3 Department of Biochemistry, School of Medicine, Case Western Reserve University, Cleveland, OH 44106, USA; 4 Childeren's Hospital Oakland Research Institute, Oakland, CA 94609, USA; 5 Department of Molecular and Structural Biochemistry, North Carolina State University, Raleigh, NC 27609-7622, USA

## Abstract

Metal ion binding was previously shown to destabilize IRE-RNA/IRP1 equilibria and enhanced IRE-RNA/eIF4F equilibria. In order to understand the relative importance of kinetics and stability, we now report rapid rates of protein/RNA complex assembly and dissociation for two IRE-RNAs with IRP1, and quantitatively different metal ion response kinetics that coincide with the different iron responses *in vivo*. k_on_, for FRT IRE-RNA binding to IRP1 was eight times faster than ACO2 IRE-RNA. Mn^2+^ decreased k_on_ and increased k_off_ for IRP1 binding to both FRT and ACO2 IRE-RNA, with a larger effect for FRT IRE-RNA. In order to further understand IRE-mRNA regulation in terms of kinetics and stability, eIF4F kinetics with FRT IRE-RNA were determined. k_on_ for eIF4F binding to FRT IRE-RNA in the absence of metal ions was 5-times slower than the IRP1 binding to FRT IRE-RNA. Mn^2+^ increased the association rate for eIF4F binding to FRT IRE-RNA, so that at 50 µM Mn^2+^ eIF4F bound more than 3-times faster than IRP1. IRP1/IRE-RNA complex has a much shorter life-time than the eIF4F/IRE-RNA complex, which suggests that both rate of assembly and stability of the complexes are important, and that allows this regulatory system to respond rapidly to change in cellular iron.

## INTRODUCTION

**Figure 1.**
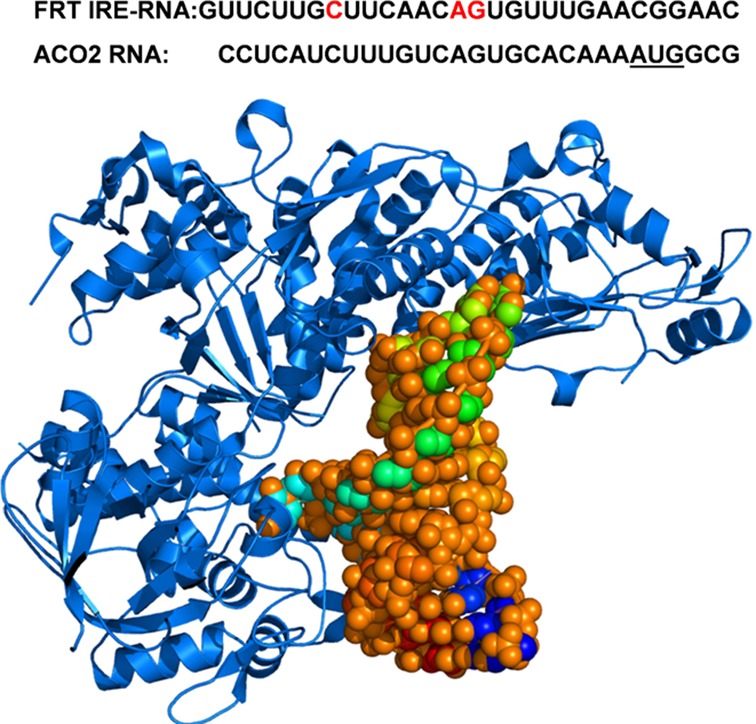
IRP1 binding to FRT IRE-RNA. In the figure, the RNA helix (space-filling model) from RNA/protein crystal structures (32) has bulge bases C8 and triloop bases A15 and G16 flipped out of the helix and making deep contacts in protein pockets; A15 and G16 involve some of the same protein contacts as the [4Fe-4S] cluster in the non-IRE binding/cytoplasmic aconitase fold of the protein. Phylogenetically conserved IRE-RNA sequences for FRT-IRE and ACO2 IRE RNAs are shown above the structure. Bases in red are flipped out from stacked (A15, G16) or disordered (C8) configurations, based on solution NMR of the free FRT RNA (37). AUG, underlined, in the ACO2 IRE-RNA is the translation initiator codon. FRT IRE-RNA is the evolutionary progenitor IRE found in lower invertebrates, while ACO2 appeared first in chordates (10). The figure was graciously prepared by Dr Suranjana Haldar who used PDB 2IPY and PYMOL.

Iron responsive elements (IREs) are cis-acting mRNA stem-loop structures that specifically bind cytoplasmic iron regulatory proteins (IRP1, IRP2) (1–4). An IRE is a ∼30 nucleotide structure folded into two RNA helices that are separated by a mid helix bulge cytosine residue and by a six nucleotide loop of the sequence 5′-CAGUGX-3′ (C-G triloop pair and X is usually a pyrimidine) (2,5–9) (Figure 1). The two IRPs, which are highly conserved themselves, bind IRE-RNA structures in a variety of animal mRNAs that appeared at various times during evolution (10) and have been extensively characterized (11–15). The name IRE (iron responsive element) was developed based on effects of increasing iron in animals or in cultured cells of animals on IRE-mRNA translation or degradation (11,12); IRE-RNA regulating translation is in the 5′ noncoding (UTR) region of the mRNAs. Iron regulatory effects measured were entry of IRE-RNA into polysomes and protein accumulation and more recently changes in RNA mobility with cell extracts. Models that developed reflected iron induced protein degradation of both IRP repressors and Fe-S cluster insertion to IRP1, but there has been no mechanistic information on whether and how Fe^2+^ changed the IRE-RNA complex stability or turnover needed to free the IRE-mRNAs for translation (11–15).

The first committed step in protein synthesis is the binding of the 5' mRNA cap (m^7^GpppN, where N represents any nucleotide) to eIF4E, the small subunit of eIF4F. eIF4G, the large subunit of eIF4F, recruits additional initiation factors including eIF4A. eIF4A is the RNA-dependent ATPase that unwinds secondary structure within the 5' noncoding region to allow 40S ribosomal scanning. Noncoding mRNA structure can make a major contribution to protein synthesis rates and gene expression in eukaryotic cells. An example is the ability of IRE-RNA to bind, competitively, two regulatory proteins: IRE-RNA binds both the generic protein synthesis enhancer, eIF4F (16), and a specific protein synthesis inhibitor, IRP (17).

Recently, we observed (16,17) metal ions (Fe^2+^ and Mn^2+^) binding to IRE-RNA, which decreased IRP1/IRE-RNA binding and increased protein synthesis initiation factor eIF4F/IRE-RNA binding. Our working model (16) suggested that eIF4F competes with IRP1 for IRE-RNA binding. Metal ions directly modulate the function of many RNA classes, e.g. rRNA (18), tRNA (19,20), ribozymes (21–25) and riboswitches in bacterial mRNAs, where metals contribute both to RNA function and to metal sensing in bacteria (22,26–29), and possibly in hammerhead, mammalian mRNAs (30). Transition metal ion-RNA complexes involve both electrostatic and coordination complex interactions. The ferritin (FRT) IRE-RNA binds metal ions (Mg^2+^) with a 1:2 stoichiometry (31,32); Mg^2+^ also binds to many rRNAs, tRNAs, riboswitches and ribozymes. In addition, the IRE-RNA also binds shape-specific metal complexes (1,10-phenanthrolene and Ru(tpy)bpy) and other small molecules at even more specific sites (33–35).

The effect of Fe^2+^ and Mn^2+^ was much larger for FRT IRE-RNA/IRP binding than for mitochondrial aconitase (ACO2) IRE-RNA/IRP (17). All IRE-RNAs share loop sequences and a stem bulge but have primary sequences and base pairs that are specific to each IRE-mRNA (Figure 1). Biologically, manganese homeostasis may have crossover points with iron homeostasis, exemplified by DMT 1 transport of both Mn^2+^ and Fe^2+^, in animals and yeast (36). We use Mn^2+^ as a biochemical model for air-sensitive Fe^2+^.

FRT IRE-RNA conformation changes when IRP1 binds, based on comparison of solution nuclear magnetic resonance (NMR) of the free IRE-RNA, the crystal structure of the FRT IRE-RNA/IRP complex (32,37) and fluorescence of 2-aminopurine IRE-RNA (16). In the IRE-RNA terminal loop, for example, conserved tri-loop bases A^15^ and G^16^ and helix bulge base C^8^ are flipped out and a large surface of the IRE-RNA remains exposed in the RNA protein complex (Figure 1), even though IRP ‘footprints’ indicate protection of the entire IRE-RNA (38); IRE-RNA folding must create ‘protected’/solvent inaccessible regions of the IRE-RNA structure. Important as RNA/protein binding equilibria are, cells must respond rapidly to changes in metabolism and the environment, which makes IRP1/IRE-RNA turnover kinetics likely to be a more sensitive regulatory target. Recently (16), we have shown that eIF4F competes with binding of IRP1 to IRE-RNA. The ability to form a repressor (IRP1/IRE) or activator (eIF4F/IRE) complex will be influenced by the kinetics of protein binding and how that binding is affected by metal ions. We now report the kinetics of IRE-RNA binding to both IRP1 and eIF4F. At low metal ion concentration, IRP1/IRE-RNA binding is fast and turnover of the complex is rapid as compared to eIF4F/IRE-RNA. At higher Mn^2+^, eIF4F association rates increased relative to IRP1. The IRP1/IRE-mRNA complex had a much shorter life-time than the eIF4F/IRE-mRNA suggesting IRP1 responds more quickly to metal ion concentrations and eIF4F/IRE stability is important for assembly of the pre-initiation complex.

## MATERIALS AND METHODS

### Preparation of binding protein and RNA

Isolation of recombinant rabbit IRP1 from yeast used methods previously described in (39), except that washed cells grown in minimal medium with 2% (w/v) dextrose were inoculated into medium containing 2% (w/v) raffinose, and then induced for 16 h in a large volume of medium with 2% (w/v) raffinose and 2% (w/v) galactose. Only a Ni-chelate column was used to purify the protein, eliminating the heparin-agarose step used before, and recombinant IRP1 was dialyzed against 20 mM Tris pH 7.9, 50 mM KCl, 1 mM EDTA with 10% (v/v) glycerol and 2% (v/v) 2-mercaptoethanol added for storage. Based on binding stoichiometry and known IRE-RNA concentrations ∼85% of the IRP protein was active in IRE-RNA binding. eIF4F was purified from rabbit reticulocyte lysate as described previously (40).

Fluorescein labeled RNA oliogonucleotides (^FI^IRE-RNA), ferritin IRE-RNA and mitochondrial aconitase IRE-RNA were purchased from Genelink (Hawthorne, NY, USA) and, after dissolving, were melted and reannealed as described in (17,41), by heating, in 40 mM HEPES/KOH, pH 7.2, to 85°C for 15 min with slow cooling to 25°C. The concentrations of FRT IRE-RNA and ACO2 IRE-RNA were determined spectrophotometrically by measuring the absorbance at 260 nm and using the absorbance of 40 μg/ml RNA as equal to 1 A_260_/ml. The concentration of protein was determined by a Bradford assay with bovine serum albumin as standard (42) using a Bio-Rad protein assay reagent (Bio-Rad Laboratories, CA, USA).

### Stopped-flow anisotropy measurements

Stopped-flow anisotropy measurements for the binding of ^FI^IRE-RNA and IRP1 protein were performed on an OLIS RSM 1000 stopped-flow system with a 1-ms dead time. The excitation and emission wavelengths for fluorescein labeled FRT IRE-RNA and ACO2 IRE-RNA (^FI^IRE-RNA) were 490 nm and 520 nm, respectively. The temperature of the flow-cell and solution reservoir was maintained at 25°C unless otherwise stated. IRP1 binding induced an increase in ^FI^IRE-RNA anisotropy. After rapid mixing of 0.1 μM (0.05 μM final) ^FI^IRE-RNA with varying concentrations (0.05 μM–1 μM final) of IRP1 protein, the time course of the anisotropy change was recorded by computer data acquisition. All measurements were performed in titration buffer, 40 mM HEPES/H^+^, pH 7.2, 1 mM MgCl_2_, 100 mM KCl, 5% (v/v) glycerol and 2% (v/v) 2-mercaptoethanol. In each experiment, 1000 pairs of data were recorded and sets of data from 5 to 7 shots were averaged to improve the signal-to-noise ratio. Each averaged set of stopped-flow anisotropy data was then fitted to nonlinear analytical equations using Global analysis software provided by OLIS.

For Mn^2+^ effects, MnCl_2_ was added to both RNA and protein solutions, at the same concentration, and the solutions were incubated separately for 15 min before adding to titration buffer containing the same metal ion concentration as the RNA and protein solutions. Experiments were performed as described above. Data were evaluated by fitting to the single and double exponential functions as described previously (43) and further analyzed as described below.

### Stopped-flow fluorescence measurements

As described elsewhere (44,45) RNA binding induced a decrease in initiation factor fluorescence. eIF4F was excited at 295 nm and the fluorescence (voltage) was measured after passing a 324 nm cut-on filter. A reference photomultiplier was used to monitor fluctuations in the lamp intensity. The temperature of the flow cell and solution reservoir was maintained using a temperature controlled circulating water bath. FRT IRE-RNA binding induced a decrease in eIF4F fluorescence. After rapid mixing of 0.1 μM (final) eIF4F with 0.1, 0.2 and 0.5 μM (final) of FRT IRE-RNA, the time course of the fluorescence intensity change was recorded by computer data acquisition. Fluorescence measurements from 5 to 7 shots were averaged to optimize the signal-to-noise ratio. Data were evaluated by fitting to the single- and double-exponential functions as described previously (46,47). We further observed the effects of Mn^2+^ (5, 25 and 50 μM final) on the binding rates of eIF4F (0.1 μM final) to FRT IRE-RNA (0.1, 0.2 and 0.5 μM final) was carried out under the same conditions as described above.

### Measurements of dissociation rate constants

To measure the dissociation rate constants of the pre-formed IRE-RNA/IRP1 complexes, RNA and protein mixtures were incubated in titration buffer for 15 min at 25°C to ensure complex equilibrium. Dissociation of pre-formed IRE-RNA/IRP1 complex was followed by measuring the decrease in anisotropy of ^FI^IRE-RNA when equal volumes of the ^FI^IRE-RNA/IRP1 complex and buffer alone or buffer containing 50 μM Mn^2+^ were mixed in the stopped-flow cell. The concentrations of the FRT IRE-RNA or ACO2 IRE-RNA and IRP1 protein in the solution were 0.05 μM and 1 μM, respectively, after mixing. The dissociation rates were determined for the relaxation experiment from the fits of the nonlinear analytical equations using Global analysis software provided by OLIS.

### Analysis of stopped-flow kinetic data

Stopped-flow data for the binding of FRT IRE-RNA or ACO2 IRE-RNA with IRP1 protein were analyzed using Global analysis software as described previously (43). Data from the anisotropy experiments were fitted to the single- and double-exponential functions. Fitted curves correspond to the following single-exponential equation

(1) \begin{equation*} {\it r}_{\it t} = \Delta {\it r}\,\exp ( - {\it k}_{{\rm obs}} .{\it t}) + {\it r}_{\rm f} \end{equation*}

where *r_t_* is the observed anisotropy at any time, *t*, and *r*_f_ is the final value of anisotropy, Δ*r* is the amplitude and *k*_obs_ is the observed first order rate constant. The double-exponential equation is

(2) \begin{equation*} {\it r}_{\it t} = \Delta {\it r}_1 \,\exp ( - {\it k}_{{\rm obs}1} .\,{\it t}) + \Delta {\it r}_2 \,\exp ( - {\it k}_{{\rm obs}2} .{\it t}) + {\it r}_{\rm f} \end{equation*}

where Δ*r*_1_ and Δ*r*_2_ are the amplitudes for the first and second components of the reaction with observed rate constants *k*_obs1_ and *k*_obs2_, respectively. The residuals were measured by the differences between the calculated fit and the experimental data. The observed rate constants were further analyzed as relaxation experiments to give *k*_on_ and *k*_off_ from the equation *k*_obs_ = *k*_on_ [IRP1] + *k*_off_. A plot of *k*_obs_ versus [IRP1] at varying IRP1 concentrations was used to give *k*_on_ (slope) and *k*_off_ (intercept). For dilution experiments of the IRE-RNA/IRP1 complex, the *k*_off_ value was obtained from the previously determined *K*_eq_ values and using *K*_D_ = *k*_off_/*k*_on_ gives *k*_obs_ = *k*_off_ ([IRP1]/*K*_D_ +1). The life-time for the eIF4F/FRT IRE-RNA and IRP1/FRT IRE-RNA complexes were calculated using equation (48), 1/τ = *k*_on_ [IRE-RNA_final_ + Protein_final_] + *k*_off_.

## RESULTS

### IRP1 associates about eight times faster with ferritin IRE-RNA than with mitochondrial aconitase IRE-RNA

**Figure 2.**
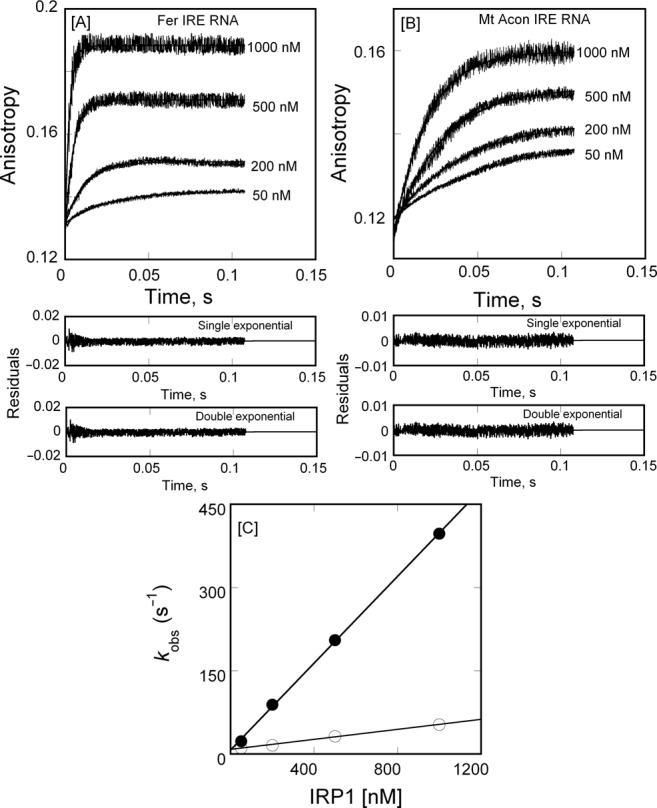
IRP1 binds IRE-RNA in a one-step, bimolecular reaction. Varying concentrations (0.05, 0.2, 0.5 and 1 μM final) of IRP1 were mixed with 50 nM (final) of (**A**) ^FI^FRT IRE-RNA and (**B**) ^FI^ACO2 IRE-RNA in 40 mM HEPES/H+, pH 7.2, 100 mM KCl, 5% glycerol and 2% 2-mercaptoethanol at 25°C. Excitation and emission wavelength were 490 nm and 520 nm, respectively. The **—** represents the fitted curve for a single-exponential function. Residuals for the corresponding single- and double-exponential fits at 1000 nM of IRP1 are shown in the lower panels. (**C**) The observed rate constant for the anisotropy change of FRT IRE-RNA (—•—) and ACO2 IRE-RNA (—○—) is plotted as a function of increasing concentrations of IRP1 protein. Data points in the plot of *k*_obs_ versus IRP1 concentration were obtained from three independent experiments and the average value of the experimental data is reported. Extrapolation (see the text) gives *k*_off_.

FRT and ACO2 IRE-RNA bind IRP1 with different affinities, based on our earlier solution and gel shift studies as well as gel shift studies of others (15,17,31,32). The IRP1 binding affinity for FRT IRE-RNA was higher than ACO2 IRE-RNA. The stopped-flow anisotropy data (Figure 2) show that the *k*_on_ of FRT IRE-RNA is about 8-fold faster than the *k*_on_ of the ACO2 IRE-RNA binding to IRP1. Data for the binding of ^FI^IRE-RNA to IRP1 were plotted as anisotropy versus time (Figure 2 A and B). The residuals representing the deviation between the calculated and experimental data indicate that the single-exponential function fits the points over the entire time course of the measurements. Further, the concentration dependence of the reaction rate was determined to distinguish between a single, bimolecular, binding step and a more complex mechanism such as a fast binding followed by a conformational change. The data fit a one-step reaction mechanism described in Equation (3), where *k*_on_ and *k*_off_ are the association and dissociation rate for the interactions of IRP1 with IRE-RNA.

(3) \begin{equation*} \text{IRP} + \text{IRE-RNA}\mathop \rightleftharpoons \limits_{\text{k}_{\text{off}} }^{\text{k}_{\text{on}} } \text{IRE-RNA} \bullet \text{IRP} \end{equation*}

The observed rate constant (*k*_obs_) is predicted to be a linear function of IRP1 concentration as shown in Equation (4),

(4) \begin{equation*} {\it k}_{{\rm obs}} = {\it k}_{{\rm on}} [{\rm IRP}1] + {\it k}_{{\rm off}} \end{equation*}

where the rates of fluorescence anisotropy change for FRT or ACO2 ^FI^IRE-RNA increased with increasing IRP1 concentrations (Figure 2A and B). The linear plot of [IRP1] versus *k*_obs_, shown in Figure 2C, was used to obtain values of *k*_on_ and *k*_off_ from the slope and the *y*-intercept, respectively. The *k*_on_ for FRT IRE-RNA was about 8-fold faster than the *k*_on_ for ACO2 IRE-RNA binding to IRP1 (Table 1).

**Table 1.** Ferritin IRE-RNA binds IRP1 faster than mitochondrial aconitase IRE-RNA

	FRT IRE-RNA	ACO2 IRE-RNA
*k*_on_ (μM^−1^ s^−1^)	400 ± 7.3	51.5 ± 1.8
*k*_off_ (s^−1^)	6.2 ± 0.3	7.0 ± 0.4
*K*_d_ (nM)^a^	15.5 ± 0.5	136 ± 2.9
*K*_d_ (nM)^b^	14.2 ± 0.3	129 ± 3.3

^a^*k*_off_/*k*_on._

^b^*K*_d_ values from equilibrium measurements (17). Errors are standard deviation (SD).

### The dissociation rate, *k*_off_, for IRP1 was similar for ferritin and mitochondrial aconitase IRE-RNAs

The *k*_off_ values for the binding of the two IRE-RNAs with IRP1 showed little difference (Table 1), which contrasts with the *k*_on_ rate. The *K*_d_ values, calculated using the equation, *K*_d_ = *k*_off_/*k*_on_, showed a 9-fold greater affinity for FRT IRE-RNA (calculated *K*_d_ = 15.5 ± 0.5 nM) compared to ACO2 IRE-RNA (calculated *K*_d_ = 136 ± 2.9 nM) with IRP1. These results are consistent with the FRT and ACO2 IRE-RNA binding to IRP1 experiments in the fluorescence steady-state, where binding differed by 9-fold. The *K*_d_ values calculated from the kinetic constants computed here agree well with the *K*_d_ value obtained by steady-state equilibrium method (17).

### Mn^2+^, a transition metal and Fe^2+^ surrogate, decreases association rates and increases dissociation rates of ferritin and mitochondrial aconitase IRE-RNAs with IRP1

**Figure 3.**
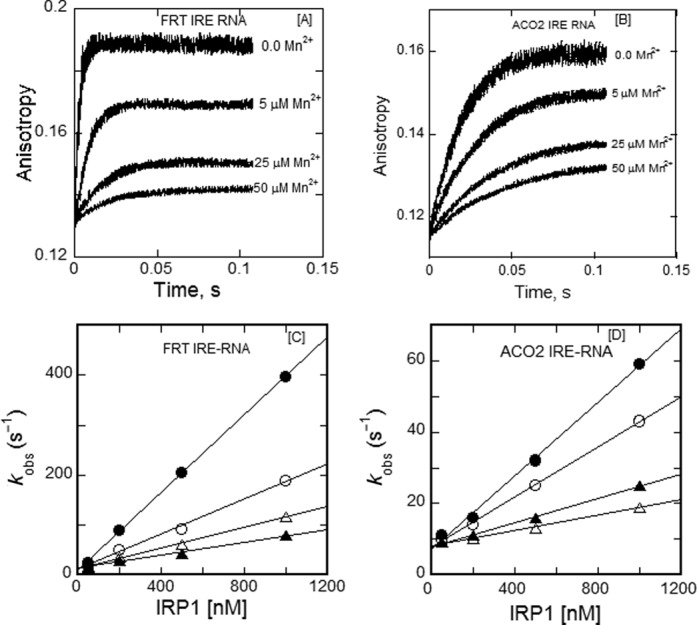
Kinetics of IRP1 induced FRT IRE-RNA-Mn^2+^ and ACO2 IRE-RNA-Mn^2+^ binding. Kinetic trace for the binding of (**A**) FRT IRE-RNA and (**B**) ACO2 IRE-RNA with IRP1 at varying concentration (5, 25 and 50 μM final) of Mn^2+^. FRT and ACO2 IRE-RNA concentrations were 50 nm (final) and the IRP1 concentration was 1 μM (final). Other conditions were as described in Figure 2. Dependence of *k*_obs_ on IRP1 concentration for the binding of FRT or ACO2 IRE-RNA as a function of Mn^2+^ concentration obtained in experiments similar to those shown in Figure 2 at the indicated Mn^2+^ concentrations, 0.0 (—•—), 5 μM (—○—), 25 μM (—▴—) and 50 μM (—Δ—), is plotted for (**C**) FRT IRE-RNA and (**D**) ACO2 IRE-RNA with IRP1. Data were fit with Equation (4). Data points in the plot of *k*_obs_ versus IRP1 concentration were obtained from three independent experiments and the average value of the experimental data is reported. The *k*_on_, *k*_off_ and *K*_d_ values at different Mn^2+^ are shown in Table 3.

In equilibrium measurements, we observed that metal ions destabilized the IRE-RNA/IRP1 complex (17). Kinetic data now show that the major effect of metal ions is on the association rates. Kinetic plots for the effect of Mn^2+^ on the binding kinetics of FRT IRE-RNA and ACO2 IRE-RNA with IRP1 are shown in Figure 3A and B; Mn^2+^ was used, as before, as an metal ion which could be studied in air and had effects similar to Fe^2+^, which had to be studied anerobically (17). Plots of the observed rate constant versus IRP1 concentration are shown for FRT and ACO2 IRE-RNAs in the presence of Mn^2+^ (Figure 3C and D). In the presence of 50 μM Mn^2+^, the association rate constant (*k*_on_) for the binding of FRT and ACO2 IRE-RNA with IRP1 decreased 6.2- and 4.8-fold, respectively. In addition, metals ions increased the dissociation (*k*_off_) rate of IRP1 for FRT IRE-RNA 2-fold and 1.1-fold increase for ACO2 IRE-RNA (Table 3). The combined effects of Mn^2+^ on the IRP1 association and dissociation rates were larger for the FRT IRE-RNA (Table 2), which parallels the selective effects of metal ions on equilibrium measurements of IRP binding to the two IRE-RNAs we observed previously (17).

**Table 2.** Mn^2+a^ slows IRP binding to and increases IRP dissociation from IRE-RNAs

	FRT IRE-RNA-Mn^2+^	FRT IRE-RNA Mn/control^c^	ACO2 IRE-RNA-Mn^2+^	ACO2 IRE-RNA Mn/control^c^
*k*_on_ (μM^−1^ s^−1^)	65 ± 2.7	0.20	10.7 ± 0.7	0.21
*k*_off_ (s^−1^)	12 ± 0.5	2.0	8.0 ± 0.4	1.1
*K*_d_ (nM)^b^	227	15.9	737	5.7

^a^(50 μM).

^b^*K*_d_ values obtained from equilibrium measurements (17) were similar to those computed from the *k*_on_ and *k*_off_ values.

^c^Ratio of value in the presence of Mn^2+^/value in the absence of Mn^2+^. Errors are SD.

**Table 3.** Mn ^2+^ slows IRP binding to and increases IRP dissociation from IRE-RNAs

Mn^2+^ μM	*k*_on_		*k*_off_		*K*_d_^a^	
	FRT IRE-RNA	ACO2 IRE-RNA	FRT IRE-RNA	ACO2 IRE-RNA	FRT IRE-RNA	ACO2 IRE-RNA
	(μM^−1^ s^−1^)		(s^−1^)		(nM)	
0	400 ± 7.3	51.5 ± 1.8	6.2 ± 0.3	7.0 ± 0.4	15.5 ± 0.5	136 ± 2.9
5	176 ± 4.3	35.2 ± 1.6	9.7 ± 0.4	7.6 ± 0.3	55 ± 2.7	217 ± 7.6
25	104 ± 3.5	17 ± 1.2	11.7 ± 0.3	7.8 ± 0.3	112.5 ± 4.1	458 ± 9.4
50	65 ± 2.7	10.7 ± 0.7	12.0 ± 0.5	8.0 ± 0.4	185 ± 6.6	727 ± 12

^a^*K*_d_ value calculated from *k*_off_/*k*_on_.

**Figure 4.**
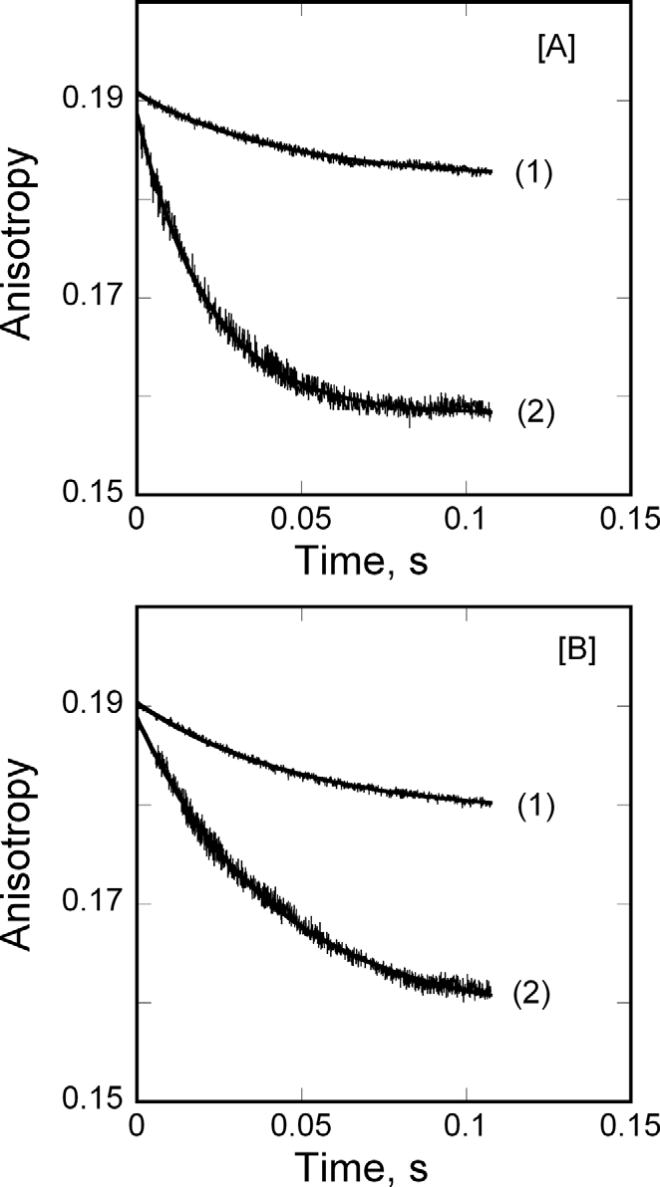
Mn^2+^ Inhibits IRE-RNA/IRP binding by changing *k*_on_ and *k*_off_ (**A**) FRT IRE-RNA IRP1 and (**B**) ACO2 IRE-RNA?IRP1 complex was diluted in the stopped-flow cell with an equal volume of titration buffer alone, curve (1) and (2) 50 μM MnCl_2_ in titration buffer. The traces were best fit with the single-exponential function. The concentrations of FRT IRE-RNA and ACO2 IRE-RNA were 50 nM, and IRP1 was 1 μM after mixing. Data were averaged from three independent experiments. The *k*_off_ value was obtained from the previously (17) determined *K*_eq_ values and using *K*_D_ = *k*_off_/*k*_on_.

The effect of Mn^2+^ on dissociation of pre-formed FRT and ACO2 IRE-RNA/IRP1 complexes was explored, by measuring the anisotropy of the relaxation reaction when equal volumes of either FRT IRE-RNA/IRP1 or ACO2 IRE-RNA/IRP1 were rapidly mixed with 50 μM Mn^2+^ in the solution buffer. The kinetic traces of the dissociation reactions initiated by the 2-fold dilution followed single-exponential kinetics (Figure 4). The dissociation rates for the two IRE-RNA/IRP1 complexes obtained by diluting with buffer in the absence of Mn^2+^ were smaller and similar to each other for the two IRE-RNAs (*k*_off_ = 8.3 ± 0.4 s^−1^ for FRT and 9.1 ± 0.6 s^−1^ for ACO2 IRE-RNA/IRP1 complex) and to the dissociation rate determined from concentration-dependent reaction kinetics (*k*_off_ = 6.2 ± 0.3 s^−1^ for FRT and 7.0 ± 0.4 s^−1^ for ACO2 IRE-RNA/IRP1 complex). In the presence of Mn^2+^, the dissociation rates for FRT (*k*_off_ = 24 ± 1.5 s^−1^) and ACO2 (*k*_off_ = 16.4 ± 1.3 s^−1^) IRE-RNA/IRP1 complexes increased 3-fold and 2-fold, respectively. Thus, Mn^2+^ can selectively increase the dissociation rates of RNA/protein complexes. Since the same protein has different dissociation rates with two IRE-RNAs (Figure 4), and since there is evidence that metal ions bind to the free IRE-RNA, but not to the free IRP1 protein, the data (Figure 4) suggest that the metal ion binding site is accessible on the IRE-RNA in the protein/RNA complex (Figure 1).

### K^+^ can partially substitute for Mn^2+^ at a concentration 6000-fold higher

**Figure 5.**
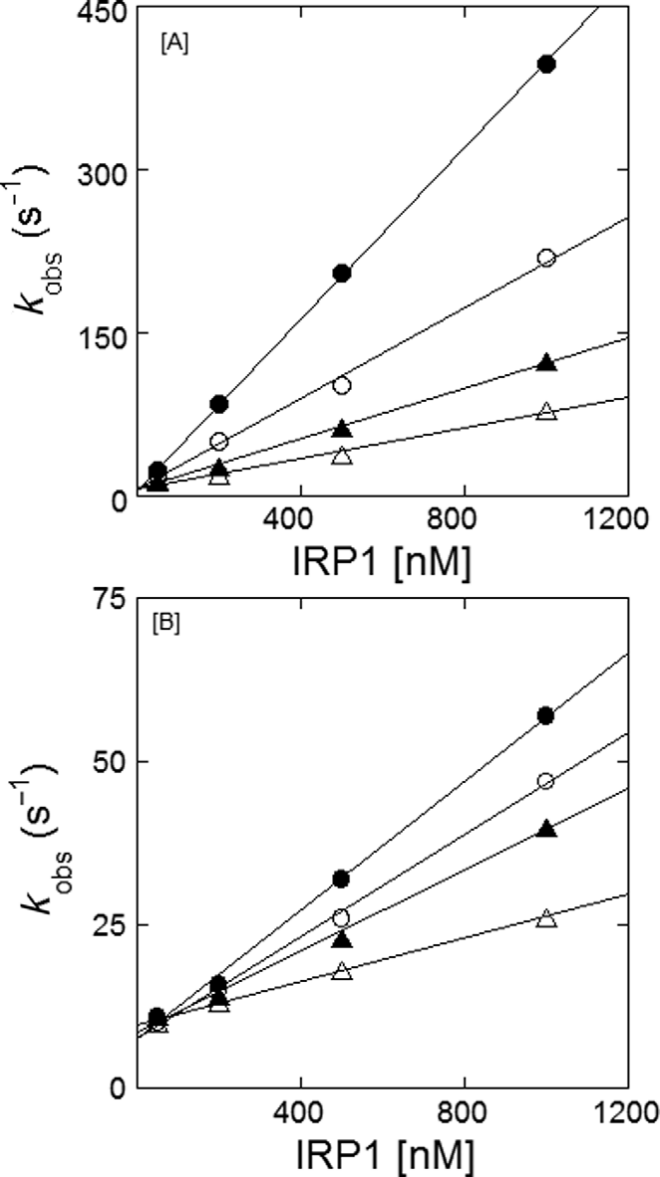
Ionic strength (K^+^) effects the kinetic rates for the binding of IRE-RNA/IRP. Dependence of *k*_obs_ on IRP1 concentration for the binding of FRT or ACO2 IRE-RNA as a function of KCl concentration obtained in experiments similar to those shown in Figure 2 at the indicated KCl concentrations, 100 mM (—•—), 150 mM (—○—), 200 mM (—▴—) and 300 mM (—Δ—), is plotted for (**A**) FRT IRE-RNA and (**B**) ACO2 IRE-RNA with IRP1. Data were fit with Equation (4). The experimental conditions were the same as described for Figure 2 except at different ionic strength. Data points in the plot of *k*_obs_ versus IRP1 concentration were obtained from three independent experiments and the average value of the experimental data is reported. The *k*_on_, *k*_off_ and *K*_d_ values at different ionic strength are shown in Supplementary Table S1.

**Figure 6.**
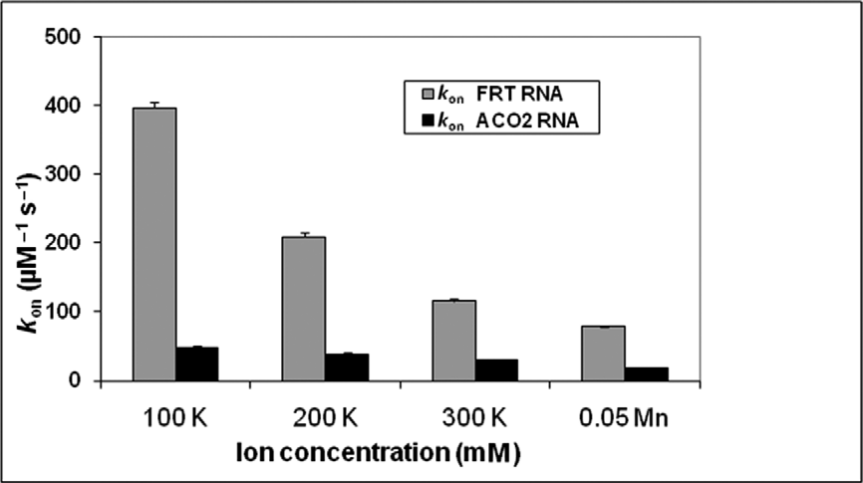
Metal effects on IRE-RNA/IRP binding kinetics contribute more to specific interactions than to electrostatics: [K^+^]/[Mn^2+^] ∼6000 for similar effects. Values for *k*_on_ in the presence of K^+^ or Mn^2+^ were compared. Very high K^+^ concentrations are required to achieve effects comparable to Mn^2+^. Data were averaged from three independent experiments; K^+^ at 100 and 200 mM are significantly different (*P* < 0.01) than 300 mM K^+^ or 0.05 mM Mn^2+^.

Electrostatic interactions with the phosphate backbone of RNA can play an important role in determining binding affinity of proteins and RNA; they are affected by the ionic strength of the environment. At high ionic strength, shielding will greatly reduce the attractive interactions. Recently, we have shown (43) that the binding affinity of initiation factors to mRNAs with internal ribosome entry sites decreases with increasing ionic strength. For the IRE-RNA/IRP interactions, we observe four effects of changing the ionic strength between 100 and 300 mM KCl: (i) The association rate (*k*_on_) decreased 7-fold for FRT IRE-RNA and 3-fold for ACO2 IRE-RNA binding to IRP1 (Figure 5 A and B, Supplementary Table S1). (ii) The *k*_off_ for IRP binding showed little change with either IRE-RNA. (iii) The ionic strength dependence of *K*_d_ values calculated from kinetic parameters (*k*_on_ and *k*_off_) at 100 mM KCl was in close agreement with the values obtained by equilibrium methods (17). For FRT IRE-RNA binding to IRP1 exhibited a *K*_d_ = 15.5 ± 0.5 nM (Table 1), and *K*_d_ = 14.2 ± 0.3 nM from steady-state equilibrium studies (17). Similarly, for ACO2 IRE-RNA binding to IRP1, the *K*_d =_ 136 ± 2.9 nM, *k*_off_/*k*_on_, again, in good agreement with the *K*_d_ = 129 ± 3.3 nM obtained by equilibrium measurements (17). (iv) *K*_d_ values for IRP binding increased about 8-fold for FRT IRE-RNA and 4-fold for ACO2 IRE-RNA over a range of KCl concentration 100–300 mM, largely due to changes in the association rate constant (Figure 6). Such results indicate a differential contribution of electrostatics to the interaction of FRT or ACO2 IRE-RNA with IRP1, possibly related to helix bending or stem interactions. However, the absence of an effect of ionic strength on dissociation (Supplementary Table S1) indicates that complex stability, which is similar for these two IRE-RNAs, is based on other non-electrostatic RNA/protein contacts, likely involving conformational change that is not rate-limiting for association.

### Kinetics of eIF4F binding to FRT IRE-RNA

**Figure 7.**
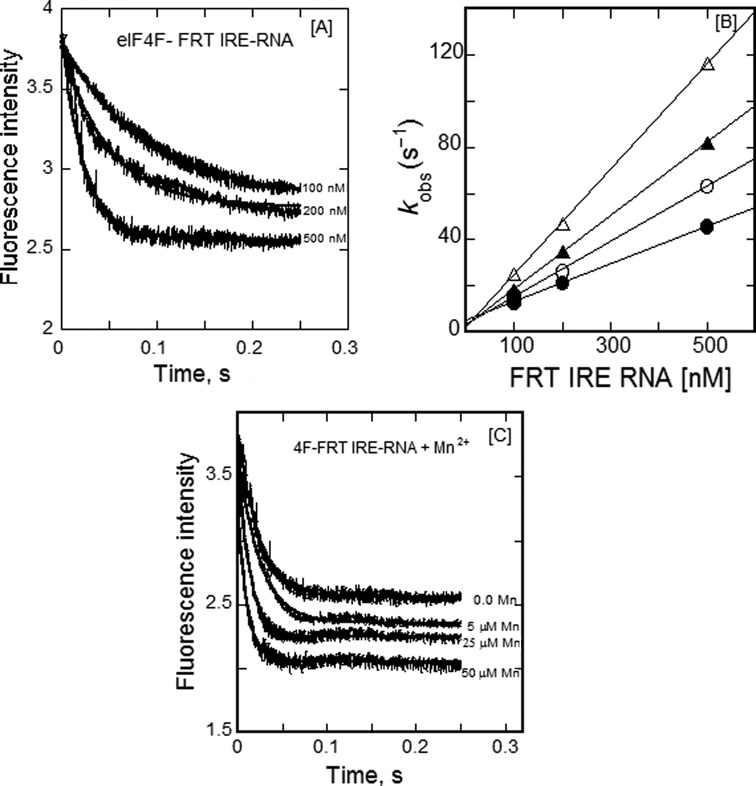
Mn^2+^ increases the kinetic rates for the binding of eIF4F with FRT IRE-RNA. (**A**) Typical time course of the intrinsic protein fluorescence intensity of eIF4F (0.1 μM final) decrease caused by binding of FRT IRE-RNA at varying concentration (0.1, 0.2 and 0.5 μM final). (**B**) The observed rate constant for the binding of eIF4F with FRT IRE-RNA is plotted as a function of the indicated Mn^2+^ concentrations, 0.0 (—•—), 5 μM (—○—), 25 μM (—▴—) and 50 μM (—Δ—). Data points in the plot of *k*_obs_ versus FRT IRE-RNA concentration were obtained from three independent experiments and the average value of the experimental data is reported. (**C**) Kinetic traces for the binding of eIF4F (0.1 μM final) with FRT IRE-RNA (0.5 μM final) in the presence of varying concentration of MnCl_2_ (5, 25 and 50 μM final). The solid line represents the fitted curve for a single-exponential function. The excitation wavelength was 295 nm. The signal represents the total fluorescence emission above 324 nm. The experimental conditions are described in Materials and Methods.

Recently, we showed (16) that eIF4F and IRP1 bind competitively to the FRT IRE-RNA. To further understand the role of eIF4F in the iron regulatory mechanism, the kinetics of eIF4F binding to FRT IRE-RNA were determined using stopped flow fluorescence measurements. We chose FRT IRE-RNA over ACO2 IRE-RNA for the kinetic studies with eIF4F because FRT IRE-RNA showed a larger effect of metal ions than ACO2 IRE-RNA. Kinetic plots for the binding of eIF4F at varying FRT IRE-RNA concentration were best fit with a single-exponential function (Figure 7A). Treatment of the data using a double-exponential fit did not improve the fit. FRT IRE-RNA concentration-dependent *k*_obs_ values obtained from kinetic rates were used for determination of *k*_on_ and *k*_off_ (Figure 7B). The observed rate constant increased linearly with an increase in FRT IRE-RNA concentration (Figure 7B). The *k*_on_ and *k*_off_ values for FRT IRE-RNA/eIF4F complex obtained from the slope and intercept were 81 ± 3.3 μM^−1^ s^−1^ and 4.8 ± 0.3 s^−1^, respectively. The life-time of the eIF4F/ FRT IRE-RNA complex and the IRP1/FRT IRE-RNA complex were calculated from the kinetic measurements as described in Materials and Methods. The life-time for the eIF4F/FRT IRE-RNA complex was 18.7 ms as compared to the life-time of IRP1/FRT IRE-RNA complex of 2.37 ms. The shorter life-time of IRP1/IRE-RNA complex is consistent with the need for rapid response to sudden change in cellular iron level. By contrast, a positive control element eIF4F/IRE-RNA may stabilize circularized mRNA for formation of the ribosome initiation complex and assembly of this complex may require a longer life-time.

### Mn^2+^ increases the association rates and decreases the dissociation rates of eIF4F/FRT IRE-RNA complex

We have previously (16) shown that Mn^2+^ increased the stability of the eIF4F/IRE-RNA complex. Here we show the effects of metal ion on the kinetic rates of eIF4F binding to FRT IRE-RNA. Kinetic plots for the binding of eIF4F with FRT IRE-RNA at different Mn^2+^ concentration are shown in Figure 7C. Plots of the observed rate constant versus FRT IRE-RNA concentration in the presence of Mn^2+^ are shown in Figure 7B. At 50 μM Mn^2+^ association rate (*k*_on_ = 228 ± 9.8 μMs^−1^) increases 3-fold and the dissociation rate (*k*_off_ = 1.8 ± 0.06 s^−1^) decreases 2.5-fold as compared to the association and dissociation rates of eIF4F/FRT IRE-RNA at zero Mn^2+^ concentration (Table 4). The *K*_d_ values, calculated using the equation, *K*_d_ = *k*_off_/*k*_on_, showed a 7-fold greater affinity for eIF4F/FRT IRE-RNA (*K*_d_ = 7.9 ± 0.3 nM) in the presence of 50 μM Mn^2+^ as compared to eIF4F/FRT IRE-RNA (*K*_d_ = 59 ± 2.4 nM) complex without metal ion. *K*_d_ values determined from the kinetics agree well with the *K*_d_ value determined from fluorescence steady-state measurements in the presence and absence of metal ion (16). Our results revealed that metal ion increased the association rates of eIF4F binding to FRT IRE-RNA, while decreasing the association rates for the IRP1 binding to FRT IRE-RNA.

**Table 4.** Mn^2+^ increases eIF4F binding to and decreases eIF4F dissociation from FRT IRE-RNAs

Mn^2+^ (μM)	*k*_on_ (μM^−1^ s^−1^)	*k*_off_ (s^−1^)	*K*_d_ (nM)^a^
0	81 ± 3.3	4.8 ± 0.3	59 ± 2.4
5	119 ± 5.4	3.3 ± 0.2	27.7 ± 1.5
25	159 ± 6.7	2.5 ± 0.1	15.7 ± 0.7
50	228 ± 9.8	1.8 ± 0.06	7.9 ± 0.3

^a^*K*_d_ value calculated from *k*_off_/*k*_on_.

## DISCUSSION

Binding of IRP1, an IRE-specific repressor, to the single IRE-RNA in the 5′ UTR of animal mRNAs inhibits translation by preventing the stable association of the small ribosomal subunit with the mRNA (49). IRE-RNA binds the translation factor eIF4F, a protein synthesis enhancer thought to be the rate limiting step in protein synthesis initiation, in addition to binding IRP1. Increase in the concentration of cellular iron in the ‘iron pool’, the labile form of cytoplasmic Fe^2+^, decreases the number of IRE-mRNA molecules bound to IRP1 and increases the number IRE-mRNA molecules bound to eIF4F (16). As a result, IRE-mRNA translation increases; coincidently proteolytic degradation of IRP and iron sulfur cluster assembly an IRP1 increases. The IRP1 binding affinities form an array with affinities differing as much as 10-fold among IRE-mRNAs (17). Thus any change in cellular iron concentration causes quantitatively different effects on each member of the IRE-mRNA family. The IRE in each IRE-mRNA has an individual sequence that is phylogenetically conserved. Ferritin IRE-RNA, the oldest IRE (10) has the most stable IRP1 binding. IRE-RNAs have specific helix sequences but share the terminal loop sequence and a helix bulge (10) (Figure 1). Fe^2+^ and Mn^2+^ destabilize of IRE-RNA/IRP1 complexes to amplify the IRE-mRNA specific binding properties and create an array of quantatively different responses to changes in environmental iron (17). Not only are the equilibrium properties of IRE-RNA/protein binding specific to each IRE-mRNA, known for decades (15,41), we now know that the kinetic binding properties of IRP1/IRE-RNA are also IRE-mRNA specific and rapid. To respond rapidly to changes in cellular Fe^2+^, repression and depression of IRE-mRNA must occur on a relatively rapid time scale. Modulation of the rates is central to iron homeostasis.

The rapidity of IRP1 binding to both FRT and ACO2 IRE-RNAs was so high it is close to diffusion limited. Difference in the very fast association rates largely accounts for the observed selective binding between FRT and ACO2 IRE-RNA. The mechanism of IRP1 binding to IRE-RNA is a simple, one-step, bi-molecular reaction that is linearly dependent on IRP1 concentration when IRP1 is in excess (Figure 2). A structural explanation for the ionic strength dependence of the IRP1 associate rate differences between FRT IRE-RNA and ACO2 IRE-RNA suggests more numerous electrostatic interactions occur in the FRT IRE-RNA/IRP1 complex than in the ACO2 IRE-RNA/IRP1 complex. However, the dissociation rates have very little ionic strength dependence which means that IRP1/IRE-RNA dissociation differs from association. Likely an additional component contributes to dissociation such as conformational change. Stability of the IRE-RNA/IRP1 complex may also be controlled by conformational changes as reported previously for protein–RNA interactions such as the ‘lure and lock’ mechanism for the binding of HuD RNA and U1A protein (43,50). For these RNA-protein interactions, initial binding is due to largely electrostatic interactions between lysine residues and the RNA phosphate groups followed by a second step of the reaction involving nearby residues that form hydrogen bonds and/or hydrophobic interactions. For IRE-RNA/IRP1 interactions, the importance of base-pair identity was indicated by base pair mutations (15) and exemplified by binding differences between ΔU^6^ FRT IRE-RNA and ACO2 IRE-RNA where only helix base pairs differ (17). Structural support for such an explanation is provided by the IRP1/FRT IRE-RNA X-ray crystal structure (32), where the upper helix in the IRE stem is distorted by a bend (∼20–30^o^) relative to the lower stem in the region of the C^8^, U^6^ bulge bases. Although similar structural information on the ACO2 IRE-RNA/IRP1 complex is currently lacking, the structure of the TfRB IRE-RNA (which like ACO2 IRE-RNA lacks the U^6^ bulge) bound to IRP1 revealed a smaller helix distortion (∼8º) (51) than that seen for the FRT IRE-RNA/IRP1 complex. This suggests that the additional U^6^ bulge in FRT IRE-RNAs confers increased flexibility between the upper and lower stems of IRE-RNAs. Both increased RNA flexibility and more electrostatic interactions between FRT IRE-RNA and IRP1 than for ACO2, likely around the C^8^ protein–RNA sites where the two IRE-RNAs differ most (Figure 1), could account for the faster association rates of FRT IRE-RNA.

Metal ions affected both the association and the dissociation rates for IRP1 binding to FRT and ACO2 IRE-RNA. The metal ion-induced decrease in IRP1/RNA association rates was larger for the FRT IRE-RNA, about 6-fold, compared to the ACO2 IRE-RNA, where the effect was about 4.8-fold reduction (Tables 2 and 3). Not only did Mn^2+^ differentially decrease IRE-RNA/IRP1 association rates, Mn^2+^ differentially increased the dissociation rates with the larger increase for FRT IRE-RNA compared to ACO2 IRE-RNA. These results suggest that the interactions with Mn^2+^ affect the intermediate conformational change in complex formation and reflect a direct sensing of the ion by IRE-RNA, analogous to the larger riboswitches. We reported earlier (17) that metal ions (5 μM Fe^2+^, Mn^2+^ and 500 μM Mg^2+^) lowered the stability of the FRT IRE-RNA/IRP1 complex; deletion of the U^6^ bulge greatly reduced the effect (17,26–29), suggesting metal binding near the C^8^ bulge and the helix bend. Metal ions not only neutralize negative charges and reduce the phosphate repulsion, transition metal ions including Fe^2+^, the physiological signal, form specific coordination complexes with bases that change conformations in the region of both the C bulge and the hairpin loop (16). Structural changes in the C bulge region also change IRE-RNA *T*_m_, nuclease sensitivity and translation efficiency and affect the hairpin loop as well (31). Such observations are consistent with an induced fit model requiring a dynamic bending of the RNA and further suggest that the region near the U^6^ may be a metal ion binding site.

Metal ion destabilization of messenger IRE-RNA/IRP1 complexes competes with the stabilization conferred by the very large number of bonds between the protein and the RNA and the stability of the RNA fold. Selective, metal-induced destabilization of FRT and ACO2 IRE-RNA/IRP1 complexes as well as other selective, metal-RNA interactions (17,26–29), emphasize the sensitivity of RNA structure/function to the environment. Based on the effects of metals on the binding kinetics observed here and on IRP1 binding equilibria (17,26–29), the conformational differences between free and bound IRE-RNA (helix bend, conformation of base in the terminal triloop and helix bulge (32,37,52)), and metal-RNA interactions (33,35), we suggest that IRE-RNA structures are very small riboswitches or riboregulators that bind Fe^2+^ within the physiological ranges of the labile iron pool concentration (53). The metal-IRE-RNA complex has a lower affinity for IRP1 (17), and faster dissociation (Figures 3 and 4) causing release of the repressor protein. The rapid release of IRP1 allows more competitive eIF binding and increased translation of the mRNA (16). Comparing the kinetics between eIF4F binding and IRP1 binding to IRE-mRNA as a function of Mn^2+^ concentration suggests that the reaction is largely kinetically controlled *in vivo* with IRP1 outcompeting eIF4F at low Fe^2+^ and the reverse being true at higher Fe^2+^ concentrations. Similarly, the longer life-time of the eIF4F/IRE-RNA complex at both high and low Mn^2+^ concentrations suggests that IRP1 responds more rapidly to cellular Fe^2+^ concentration changes while eIF4F forms a more stable platform for ribosome binding. *In vivo* life-times of the complexes will depend on the cellular concentrations of IRP and eIF4F and may be influenced by other cellular components.

Riboswitches in eubacteria frequently involve larger RNA structures that modulate translation initiation (54) and use metabolites to control the accessibility of the Shine-Dalgarno sequence and recruitment of 30S ribosomes. By analogy, we suggest that access to eIFs and ultimately 40S ribosomes for translation is controlled by the small IRE-RNA riboregulators through metal binding which influences association and dissociation of IRE-RNA/protein complexes and subsequent gene expression.

## SUPPLEMENTARY DATA

Supplementary Data are available at NAR Online.

## FUNDING

National Institutes of Health [DK20251 to D.J.G. and E.C.T., DK47281 to W.E.W., in whole or in part]; National Science Foundation [MCB 1157632 to D.J.G.]. Source of open access funding: National Science Foundation, National Institutes of Health.

*Conflict of interest statement*. None declared.

## Supplementary Material

SUPPLEMENTARY DATA
