# Supplementary material for: Rapid kinetics of iron responsive element (IRE) RNA/iron regulatory protein 1 and IRE-RNA/eIF4F complexes respond differently to metal ions
Source: Nucleic Acids Res. 2014 Apr 9;42(10):6567–77. doi: 10.1093/nar/gku248 (PMC4041422; doi:10.1093/nar/gku248)
Supplement: SUPPLEMENTARY DATA [file supp_42_10_6567__index.html]

Rapid kinetics of iron responsive element (IRE) RNA/iron regulatory protein 1 and IRE-RNA/eIF4F complexes respond differently to metal ions — Rapid kinetics of iron responsive element (IRE) RNA/iron regulatory protein 1 and IRE-RNA/eIF4F complexes respond differently to metal ions — SUPPLEMENTARY DATA 

# Rapid kinetics of iron responsive element (IRE) RNA/iron regulatory protein 1 and IRE-RNA/eIF4F complexes respond differently to metal ions

## SUPPLEMENTARY DATA

**Files in this Data Supplement:**

- Supplementary Data
