# Supplementary material for: Rapid kinetics of iron responsive element (IRE) RNA/iron regulatory protein 1 and IRE-RNA/eIF4F complexes respond differently to metal ions
Source: Nucleic Acids Res. 2014 Apr 9;42(10):6567–77. doi: 10.1093/nar/gku248 (PMC4041422; doi:10.1093/nar/gku248)
Supplement: SUPPLEMENTARY DATA [file supp_gku248_nar-00144-r-2014-File009.pdf]

Table S1. Concentrations of K^+ required to changes the binding kinetics of ferritin IRE-RNA or mitochondrial aconitase IRE-RNA to IRP1 protein.

KCl mM	k_{on} (μM^{-1} s^{-1})		k_{off} (s^{-1})		K_d[a] (nM)	
	FRT IRE -RNA	ACO2 IRE -RNA	FRT IRE -RNA	ACO2 IRE -RNA	FRT IRE -RNA	ACO2 IRE -RNA
100	400 ± 7.3	51.5 ± 1.8	6.2 ± 0.3	7.0 ± 0.4	15.5 ± 0.5	136 ± 2.9
150	209 ± 5.4	39 ± 2.2	6.3 ± 0.2	7.4 ± 0.3	30 ± 0.7	190 ± 9.0
200	116 ± 4.2	31 ± 1.4	6.8 ± 0.4	8.4 ± 0.7	58.6 ± 2.5	271 ± 12
300	57 ± 1.9	17 ± 0.9	7.2 ± 0.3	9.4 ± 0.5	126 ± 5.7	553 ± 13

[a]K_d value calculated from k_{off}/k_{on}.
